# Diagnostic Discordance Between IgM Serology and TSA-56 Real-Time Polymerase Chain Reaction (PCR) for Detecting Scrub Typhus Among Patients With Acute Undifferentiated Febrile Illness in Western Odisha

**DOI:** 10.7759/cureus.117277

**Published:** 2026-10-01

**Authors:** Rakesh K Panda, Preetinanda M. Dash, B. Maheswar Rao, Saroj R Mohanty, Ashok K Behera, Pragalbha Jaysingh, Sushree P Satapathy

**Affiliations:** 1 Microbiology, Maharaja Jajati Keshari Medical College and Hospital, Jajpur, IND; 2 Microbiology, Bhima Bhoi Medical College and Hospital, Balangir, IND; 3 Multidisciplinary Research Unit (MRU) (Department of Health Research, DHR), Bhima Bhoi Medical College and Hospital, Balangir, IND; 4 Pediatrics, Maharaja Krishna Chandra Gajapati Medical College and Hospital, Berhampur, IND; 5 Pediatrics, Bhima Bhoi Medical College and Hospital, Balangir, IND; 6 General Medicine, Bhima Bhoi Medical College and Hospital, Balangir, IND; 7 General Medicine, Srirama Chandra Bhanja Medical College and Hospital, Cuttack, IND; 8 Community and Family Medicine, Bhima Bhoi Medical College and Hospital, Balangir, IND

**Keywords:** acute undifferentiated fever, igm elisa, immunochromatographic test, orientia tsutsugamushi, real-time pcr, scrub typhus, tsa-56, western odisha

## Abstract

Background: Scrub typhus (ST), caused by *Orientia tsutsugamushi*, is an important cause of acute undifferentiated febrile illness (AUFI) in India. Diagnosis remains challenging because clinical manifestations are often nonspecific and the performance of diagnostic assays varies according to the stage of illness. Serological assays are widely used, whereas molecular assays may detect infection during the period of rickettsemia. The present study evaluated the occurrence of ST among patients with acute undifferentiated fever in Western Odisha and examined the discordance between IgM-based serology and TSA-56 real-time polymerase chain reaction (qPCR).

Methods: An observational study was conducted at Bhima Bhoi Medical College and Hospital (BBMCH), Balangir, Odisha. Patients presenting with AUFI to the Medicine and Pediatrics outpatient departments were enrolled after written informed consent. A total of 397 febrile patients were included. Serum was tested using an IgM immunochromatographic test (ICT) and IgM enzyme-linked immunosorbent assay (ELISA), while EDTA blood was used for molecular testing. DNA was extracted by a column-based method, and TSA-56 qPCR was performed using a commercial qPCR assay. Complete blood counts were also evaluated.

Results: The study population comprised 244 males (61%) and 153 females (39%), with an age range of 3-85 years and a mean age of 37.11 ± 18.41 years. ST positivity was 16.8% (67/397) by IgM ICT and 19.64% (78/397) by IgM ELISA. TSA-56 qPCR was positive in 37 of 178 tested samples (20.78%). Among the samples evaluated by both methods, 12/257 (4.6%) ELISA-negative samples were qPCR-positive, whereas 6/78 (7.7%) ELISA-positive samples were qPCR-negative. Compared with the ST-negative group, patients classified as positive had a higher mean white blood cell count (9.94 ± 3.93 vs. 7.77 ± 3.57 × 10³/µL; p = 0.002) and lower mean platelet count (160.30 ± 87.64 vs. 220.31 ± 107.88 × 10³/µL; p = 0.001).

Conclusion: The findings demonstrate clinically relevant discordance between serological and molecular detection of ST in patients with acute undifferentiated fever in Western Odisha. The presence of qPCR-positive cases among ELISA-negative patients suggests that reliance on serology alone may miss some infections, particularly during early illness. Conversely, qPCR negativity among ELISA-positive patients may reflect differences in the timing of antibody and bacterial DNA detection. A combined diagnostic approach may therefore provide more comprehensive case detection in endemic clinical settings.

## Introduction

Scrub typhus (ST) is caused by the obligate intracellular bacterium *Orientia tsutsugamushi*. It is transmitted by the larval stage of trombiculid mites and is increasingly recognized as an important cause of acute undifferentiated febrile illness (AUFI) in India. The clinical presentation is frequently nonspecific and may overlap with dengue, malaria, leptospirosis, enteric fever, and other infectious causes of fever. Consequently, laboratory confirmation is particularly important in endemic regions [1-3]

The diagnosis of ST remains challenging because no single diagnostic assay performs optimally throughout the course of infection. Serological methods, particularly IgM enzyme-linked immunosorbent assay (ELISA), are widely used because they are comparatively simple, scalable, and suitable for routine diagnostic laboratories. The 56-kDa antigen of *O. tsutsugamushi* is highly immunogenic and has been incorporated into several serological assays [1,4]. However, antibody responses may not be detectable during the earliest phase of infection. Thus, a negative serological result does not necessarily exclude infection in a patient presenting early in the course of illness [2,3].

Molecular methods provide an alternative approach because they detect bacterial DNA rather than the host immune response. Polymerase chain reaction (PCR) assays targeting the 56-kDa antigen gene, 47-kDa gene, 16S rRNA, and other genomic regions have been evaluated for the diagnosis of ST. Real-time PCR targeting the TSA-56 region is particularly attractive because it allows rapid amplification and detection of *O. tsutsugamushi* DNA. Nevertheless, molecular sensitivity can also vary according to bacterial load, timing of sampling, prior antimicrobial therapy, specimen type, and genetic diversity among circulating *Orientia *strains [2,5].

Previous Indian studies have demonstrated that serological and molecular tests do not always identify the same group of patients. Gupta et al. reported substantial differences in performance between IgM ELISA, rapid flow assays, and a nested PCR targeting the 56-kDa gene [6]. Similarly, a multicenter evaluation by Kannan et al. demonstrated that both serological and molecular methods have strengths and limitations and that test performance depends substantially on the diagnostic reference framework used [7].

Western Odisha represents an important setting for studying ST because acute febrile illnesses are frequently encountered in the region, while detailed data comparing serological and molecular detection are limited. The present study was therefore undertaken among patients presenting with acute undifferentiated fever at Bhima Bhoi Government Medical College and Hospital (BBMCH), Balangir, to determine the occurrence of ST using IgM-based assays and TSA-56 qPCR and, importantly, to characterize discordant results between serological and molecular testing.

## Materials and methods

Study design and setting

This was an observational study conducted at BBMCH, Balangir, Odisha. The study population was drawn from Balangir and neighboring districts of Western Odisha. The region has a hot and humid climate and represents an important catchment area for patients presenting with AUFI.

The study received approval from the MRU-LRAC (01, dated 10/13/2022) and the Institutional Ethics Committee of BBMCH (31/IEC, dated 07/11/2022). The project was funded by the Department of Health Research (DHR), New Delhi. Written informed consent was obtained from all participants before the collection of demographic data and clinical specimens.

The study was conducted over a period of two years, from 2022 to 2024.

As this was a hospital-based AUFI study, the inclusion and exclusion criteria for the study population were established in accordance with the guidelines recommended by the Indian Council of Medical Research (ICMR).

Study population

The study population comprised patients presenting with AUFI to the Departments of General Medicine, Pediatrics, and Emergency Medicine at BBMCH, Balangir, Odisha, from July to November.

Inclusion criteria

Patients included in the study were residents of Balangir, aged ≥2 years, who presented with fever, defined as an axillary temperature of ≥38.0°C, with a duration of 5-28 days at the time of presentation, with or without the presence of an eschar, lymphadenopathy, rash, headache, myalgia, hepatosplenomegaly, or breathlessness. AUFI was defined clinically as fever without an evident focus of infection after initial clinical assessment and preliminary laboratory evaluation. Written informed consent was obtained from all participants. For minors or patients unable to provide consent, informed consent was obtained from a legally authorized representative, with assent obtained from children aged 12-18 years. 

Exclusion criteria

Patients with fever attributable to a confirmed etiology at initial screening were excluded from the study. These included, but were not limited to, malaria diagnosed by microscopy or rapid diagnostic test; dengue confirmed by NS1 antigen or IgM ELISA; enteric fever confirmed by blood culture; bacteremia; urinary tract infection with significant bacteriuria; radiologically confirmed pneumonia; and SARS-CoV-2 infection confirmed by qPCR or rapid antigen test (RAT).

Patients with a history of prior diagnosis of ST or those who had received anti-rickettsial antibiotics, namely, doxycycline, azithromycin, or chloramphenicol, for more than 48 hours within 72 hours prior to enrollment were also excluded to avoid false-negative serological results. Patients with a history of ST infection within the preceding three months were excluded because persistent IgM antibodies may result in false-positive serological results.

Patients with autoimmune disorders or conditions known to cause false-positive IgM results, such as systemic lupus erythematosus or rheumatoid arthritis, were excluded. Patients who refused to provide informed consent or had an inadequate blood sample volume for serological testing were also excluded.

Case definition

A confirmed case of ST was defined as a patient meeting the inclusion criteria and having either a positive result for anti-*O. tsutsugamushi* IgM by IgM ELISA (Scrub Typhus Detect™, InBios International Inc., Seattle, WA, USA), with an optical density cutoff of >0.5 as per the manufacturer’s instructions, or a positive PCR result targeting the 56-kDa gene.

Sample collection and processing

Five milliliters of whole blood was collected from each participant and divided between a gel separator tube and an EDTA tube. Approximately 3 mL was collected into the gel separator tube for serum-based testing, and 2 mL was collected into the EDTA tube for hematological and molecular investigations.

Serum samples were tested for ST IgM using an immunochromatographic test (ICT) (Scrub Typhus Detect™ IgM Rapid Test) and for ST IgM antibodies by ELISA (Scrub Typhus Detect™ IgM ELISA). EDTA blood was used for complete blood count analysis (XN-350, Sysmex) and molecular diagnosis. DNA was extracted using the column-based method (Qiagen, Hilden, Germany), followed by quality assessment using 1% agarose gel electrophoresis and quantification using a DeNovix DS-11 spectrophotometer. TSA-56 qPCR was performed using a CFX96 real-time PCR system (Bio-Rad, Hercules, CA, USA) with the Geno-Sen’s ST real-time PCR kit (Genome Diagnostics, Janakpuri, New Delhi, India) (Figure 1).

**Figure 1:**
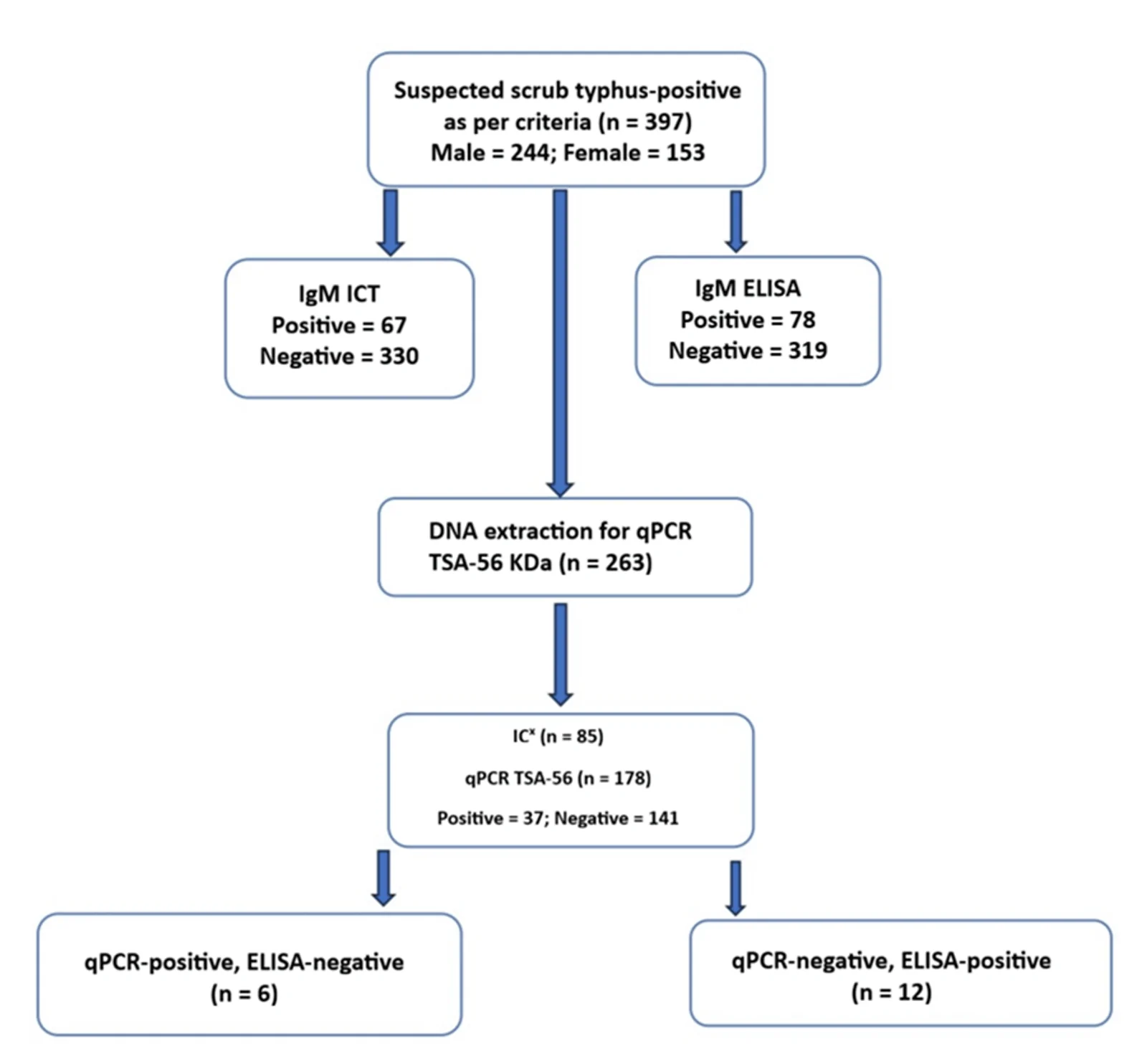
Patient flowchart as per STROBE guidelines STROBE: Strengthening the Reporting of Observational Studies in Epidemiology, ICT: immunochromatographic test, ELISA: enzyme-linked immunosorbent assay, qPCR: real-time polymerase chain reaction.

Hematological investigations

Complete blood counts were performed using an automated XN-350 hematology analyzer (Sysmex). The study specifically evaluated white blood cell and platelet counts among patients classified according to their ST test results.

Statistical analysis

All quantitative data were entered into Microsoft Excel 2016 (Microsoft Corp., Redmond, WA, USA) and analyzed using R statistical software (R Foundation for Statistical Computing, Vienna, Austria). Categorical variables were summarized as frequencies and percentages, whereas continuous variables were expressed as mean ± standard deviation. Mean white blood cell (WBC) and platelet counts were compared between the ST-positive and ST-negative groups using an independent-samples t-test with Welch’s correction (two-tailed). The diagnostic performance of the IgM ICT and IgM ELISA was evaluated using TSA-56 qPCR as the reference standard. Sensitivity, specificity, positive predictive value (PPV), negative predictive value (NPV), overall concordance (accuracy), and discordance were calculated. Agreement between the serological tests and TSA-56 qPCR was assessed using Cohen’s kappa (κ). A two-sided p-value < 0.05 was considered statistically significant. The diagnostic performance measures reported in the study were descriptive measures and, therefore, were not associated with p-values.

## Results

Demographic characteristics

The study population included 244 males (61%) and 153 females (39%), with a mean age of 37.11 ± 18.41 years. Among participants classified as ST-positive, cases were distributed across several age groups. Both males and females were represented, with positivity occurring particularly among adults in the 31-60-year age groups. The available project data showed that among positive males, the highest proportion was observed in the 41-50-year age group (24.4%), while among positive females, the 41-50-year age group accounted for 25.0% (Figure 2).

**Figure 2:**
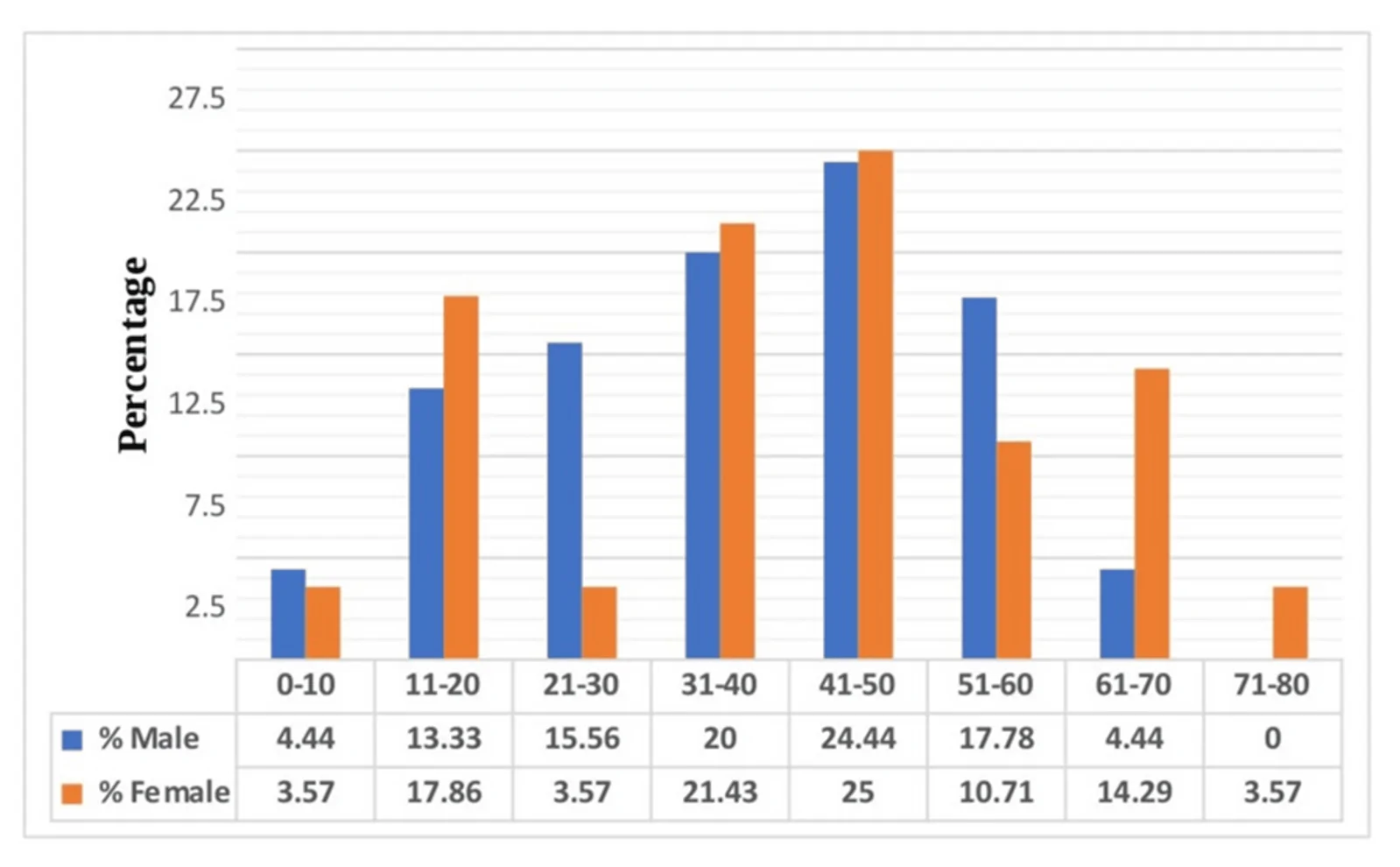
Age- and sex-wise distribution of scrub typhus-positive patients

Detection of ST by different diagnostic methods

IgM ICT identified 67 of 397 participants as positive, corresponding to an overall positivity of 16.8%. IgM ELISA identified 78 of 397 participants as positive, corresponding to a positivity rate of 19.64%. TSA-56 qPCR was positive in 37 of 178 tested samples, giving a positivity rate of 20.78% (Table 1).

**Table 1:** Positivity of different diagnostic assays for scrub typhus (ST) ICT: immunochromatographic test, ELISA: enzyme-linked immunosorbent assay, qPCR: real-time polymerase chain reaction.

Diagnostic method	Number tested	Positive	Negative	% of positivity
IgM ICT	397	67	330	16.8%
IgM ELISA	397	78	319	19.64%
TSA-56 qPCR	178	37	141	20.78%

Thus, the proportion identified as positive varied according to the diagnostic method used. ELISA identified more positive participants than ICT, while the positivity observed among the subset tested by TSA-56 qPCR was numerically higher.

Discordance between IgM ELISA and TSA-56 qPCR

A clinically important finding was the discordance between serological and molecular testing.

Among the ELISA-negative samples tested by qPCR, 12 of 257 (4.6%) were qPCR-positive. Conversely, 6 of 78 (7.7%) ELISA-positive samples were qPCR-negative (Table 2).

**Table 2:** Discordant results between IgM ELISA and TSA-56 qPCR ELISA: enzyme-linked immunosorbent assay, qPCR: real-time polymerase chain reaction.

ELISA status	qPCR result	Number	Percentage
Negative	Positive	12	4.6% of ELISA-negative samples
Positive	Negative	6	7.7% of ELISA-positive samples

These findings indicate that the two diagnostic approaches identified partially different patient groups. Importantly, the presence of qPCR positivity among ELISA-negative samples indicates that a proportion of infections may not be detected if diagnosis relies exclusively on IgM serology.

At the same time, qPCR negativity among ELISA-positive participants demonstrates that detection of bacterial DNA and detection of an antibody response do not necessarily occur concurrently. This observation is biologically plausible because bacterial DNA and antibody production may have different temporal profiles during infection [2,5,7].

Hematological findings

The ST-positive group demonstrated significant alterations in hematological parameters compared with the negative group. The mean white blood cell count was significantly higher among positive participants than negative participants (9.943 ± 3.93 vs. 7.770 ± 3.57 × 10³/µL; p = 0.002). Conversely, the mean platelet count was significantly lower in the positive group (160.301 ± 87.64 vs. 220.309 ± 107.88 ×10³/µL; p = 0.001).

Among ST-positive participants, mild anemia was the most frequent category among males, accounting for 75.6%, whereas moderate anemia was relatively more frequent among females (42.9%). Severe anemia was observed in 4.4% of positive males and 7.1% of positive females, while life-threatening anemia was reported in 3.6% of positive females (Figure 3).

**Figure 3:**
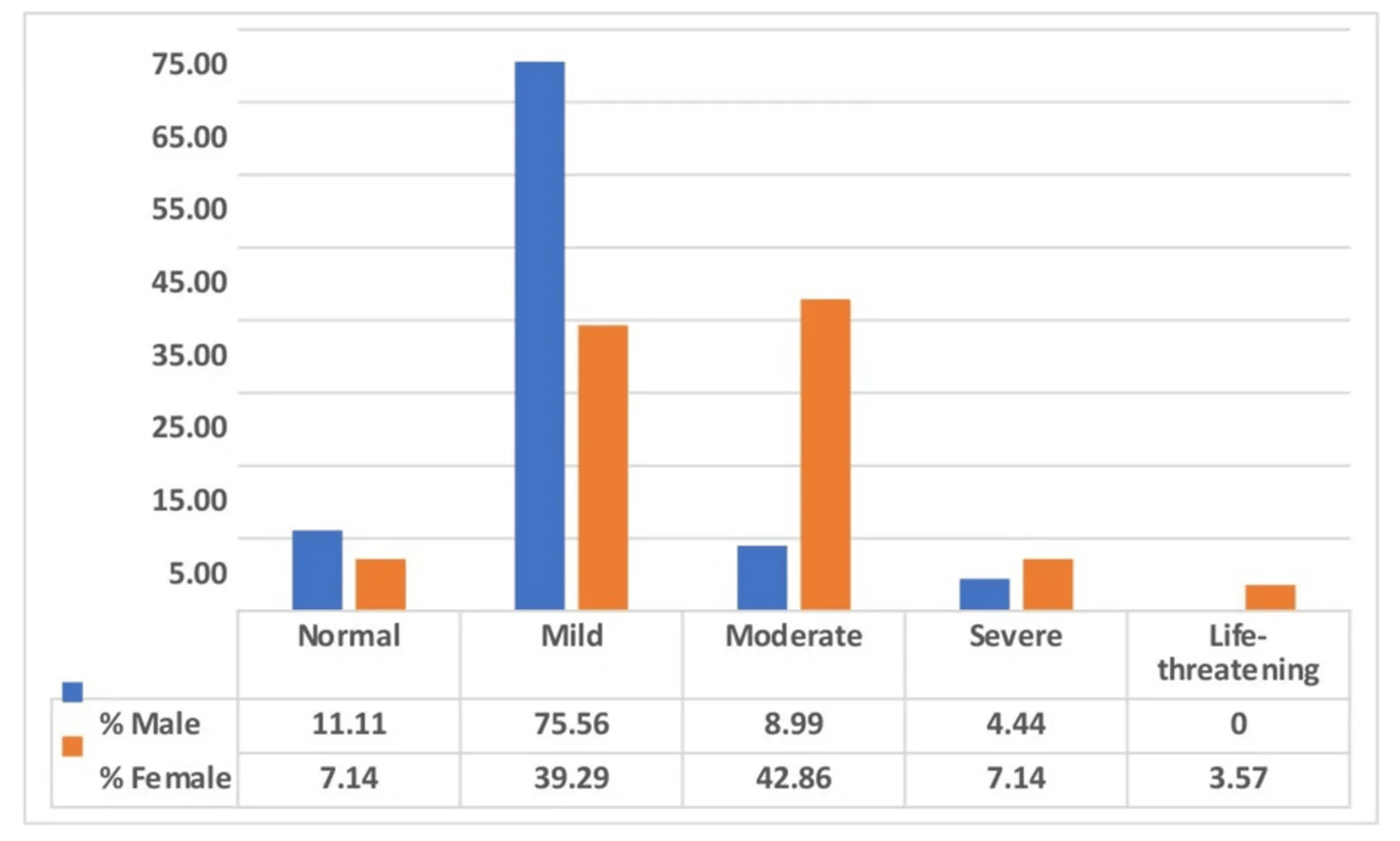
Anemia patterns among the scrub typhus-positive population

The platelet data also showed lower mean platelet counts among positive participants across several age groups, although the available presentation does not provide sufficient information to formally test age-specific differences.

Interpretation of diagnostic accuracy

In the present study, the diagnostic performance of ELISA and ICT was evaluated against TSA-56 qPCR as the reference standard in (n = 178) samples (Table 3).

**Table 3:** Diagnostic matrix

Diagnostic matrix	ICT vs TSA-56 qPCR	ELISA vs TSA-56 qPCR
Sensitivity	42.86%	71.43%
Specificity	91.75%	92.78%
Positive predictive value (PPV)	27.27%	41.67%
Negative predictive value (NPV)	95.70%	97.83%
Overall concordance	88.46%	91.35%
Discordance	11.54%	8.65%
Cohen’s kappa	0.2736	0.4823

Immunochromatographic test 

The ICT showed an overall concordance (accuracy) of 88.46%, which is slightly lower than that observed for ELISA. The discordance rate was 11.54%, indicating a higher level of disagreement with PCR results compared to ELISA.

The sensitivity of ICT was 42.86%, suggesting that the test detected less than half of the true-positive cases, thereby missing several infected individuals. This reflects a relatively low ability of ICT to identify all true-positive cases. The specificity of ICT was 91.75%, demonstrating that the test correctly classified the majority of individuals who were disease-negative. Thus, ICT also has a good ability to correctly identify non-infected individuals. Overall, ICT shows high specificity but low sensitivity, indicating that while it performs well in identifying negative cases, it is less reliable for detecting positive cases.

ELISA test

The ELISA test demonstrated an overall concordance (accuracy) of 91.35%, indicating that the ELISA results were in agreement with the PCR results in the majority of cases. The discordance rate was 8.65%, showing that a small proportion of ELISA results differed from the PCR findings. The sensitivity of ELISA was 71.43%, which means that the test was able to correctly identify approximately 71% of the true-positive cases detected by PCR. However, some positive cases were missed, resulting in false-negative results. The specificity of ELISA was 92.78%, indicating that the test correctly identified nearly 93% of individuals who were actually disease-negative according to PCR. This reflects a strong ability of ELISA to correctly classify non-infected individuals. Overall, these findings suggest that ELISA has good diagnostic performance with high specificity and moderate sensitivity, making it a reliable test for ruling out infection when the result is negative.

Therefore, ELISA appears to be the more reliable diagnostic method, while ICT may be useful as a rapid screening test but with limited ability to detect all positive cases.

## Discussion

The present study provides evidence that ST contributes substantially to the burden of AUFI in Western Odisha and demonstrates that serological and molecular assays may identify different subsets of patients. Among 397 febrile patients, IgM ICT and IgM ELISA detected ST in 16.8% and 19.64%, respectively, while TSA-56 qPCR was positive in 20.78% of the 178 patients tested by the molecular assay. The most relevant observation was the presence of qPCR positivity among ELISA-negative samples and qPCR negativity among ELISA-positive samples.

The overall positivity observed in this study is consistent with the increasing recognition of ST as an important cause of acute febrile illness in India. Diagnosis is particularly challenging in endemic areas because clinical manifestations overlap considerably with those of other infections, and characteristic findings such as an eschar may not be present in every patient [1-3].

There are very few publications comparing the three aforementioned diagnostic assessments in parallel. Some studies have compared the diagnostic performance of serological and molecular methods for ST, highlighting that the diagnostic utility of each method may vary depending on the stage of illness. Ramalingam et al. reported that IgM ELISA demonstrated the highest sensitivity (96.30%) and specificity (100%), followed by the Rapid Card test (sensitivity, 93.55%; specificity, 99.38%) and RT-PCR (sensitivity, 92.86%; specificity, 99.44%) [8]. Similarly, Elangovan et al. reported sensitivities of 98.7% and 97.4% for ST IgM ICT and IgM ELISA, respectively, with specificities of 96.3% and 95.9%, and excellent concordance between the two assays [9]. Sherawat et al. demonstrated that RT-PCR was particularly useful during the first week of fever, whereas IgM ELISA was more frequently positive during the later febrile phase, supporting a stage-specific diagnostic approach [10]. Gatika et al. similarly demonstrated complementary diagnostic performance of PCR and IgM ELISA, with molecular detection being particularly useful during the early seronegative phase and serology becoming increasingly useful later in the course of illness [11]. In contrast, Kumar et al. reported only 59% concordance between PCR and IgM ELISA, emphasizing that these tests should not necessarily be considered interchangeable and that test selection should take into account the day of illness [12].

Vanlalruati et al. documented suboptimal performance of the SD Bioline ICT, with a specificity of 65% and a sensitivity of 57%, and proposed its cautious use [13]. Anitharaj et al. reported sensitivity, specificity, PPV, and NPV of 99.25%, 93.02%, 95.68%, and 98.77%, respectively, for IgM ICT against IgM ELISA as the reference standard. Both assays were from InBios. The authors suggested that the IgM ICT, as a point-of-care (PoC) test, could be useful in both urban and remote rural areas of India [14]. In a rural setting in Rajasthan, Dashora et al. observed positivity rates of 45.83% (99/216) and 43.51% (94/216) by rapid ICT and IgM ELISA, respectively, and considered rapid ICT to have good sensitivity and specificity compared with IgM ELISA [15]. Pote et al. reported high diagnostic accuracy for IgM ELISA, with a sensitivity of 99.9% and specificity of 99.15%, compared with a sensitivity of 38% and specificity of 100% for ICT, suggesting limitations of ICT in acute serum samples [16].

IgM ELISA remains one of the most practical laboratory approaches for diagnosis because it can be standardized and applied to large numbers of samples. Previous studies have demonstrated good performance of IgM ELISA, although estimates vary according to the reference standard, assay antigen, and patient population [4,6,7]. The present study similarly found IgM ELISA to be useful for identifying a substantial proportion of febrile patients with evidence of ST infection.

However, the 12 qPCR-positive results among 257 ELISA-negative samples are particularly noteworthy. This finding suggests that molecular testing may identify infection in some patients before a detectable IgM response has developed. PCR-based assays detect pathogen DNA and may therefore be particularly useful during the early phase of illness. Existing literature supports the concept that molecular detection and antibody detection represent different biological events and that their diagnostic yields may vary according to the timing of specimen collection [2,5,7].

These findings have practical implications. In routine clinical practice, serology is often used as the principal laboratory method for ST diagnosis. However, when serological testing is performed very early in the illness, a negative result may not exclude infection. Molecular testing can provide complementary information during this phase. Conversely, once an antibody response has developed, serology may remain positive even when circulating bacterial DNA is no longer detectable. The two methods should therefore be considered complementary rather than competing diagnostic strategies.

The present observations are broadly consistent with previous comparative studies. Gupta et al. reported differences in the performance of IgM ELISA, rapid tests, and 56-kDa PCR in Indian patients and emphasized the continuing challenges in laboratory confirmation of ST [6]. Kannan et al., in a large Indian evaluation, similarly demonstrated that the diagnostic performance of serological and molecular tests differs according to the reference framework and that no single assay can necessarily be regarded as perfect [7].

Another important finding in our study was the association of ST positivity with hematological abnormalities. The positive group had a significantly higher mean white blood cell count and a significantly lower platelet count than the negative group. Thrombocytopenia is a recognized laboratory feature of ST and may provide an additional clinical clue in patients presenting with undifferentiated fever. Indian national guidance also recognizes thrombocytopenia and leukocytosis as useful supportive laboratory findings in rickettsial diseases [8]. The lower platelet count observed in the positive group is therefore clinically relevant. Nevertheless, thrombocytopenia is not specific to ST and can occur in dengue, malaria, sepsis, and other febrile illnesses. It should consequently be regarded as a supportive rather than diagnostic finding. Similarly, the higher white blood cell count observed among positive patients cannot, by itself, distinguish ST from other bacterial or inflammatory causes of fever.

An important strength of this study is that it was conducted in a real-world clinical population presenting with acute undifferentiated fever in Western Odisha. The inclusion of both serological and molecular testing provided an opportunity to identify diagnostic discordance that would remain undetected if only one method were used. The study also provides local data from a region where ST is clinically relevant, but molecular-serological comparison data remain limited.

Several limitations should be acknowledged. First, only 178 of the 397 enrolled patients underwent TSA-56 qPCR according to the available project dataset. Therefore, the molecular positivity rate should be interpreted as applying to the tested subset rather than to the entire cohort. Second, the study did not use culture isolation, paired serology, IFA, or an independent composite reference standard to establish definitive infection status. Consequently, ELISA and qPCR discordance cannot be interpreted simply as false-positive or false-negative results. Third, the study used a TSA-56 molecular target, and sequence variation within the 56-kDa gene may influence PCR detection in genetically diverse O. tsutsugamushi populations [2,5].

The study therefore supports a cautious interpretation: neither a negative IgM ELISA nor a negative TSA-56 qPCR result should be considered sufficient, in isolation, to exclude ST in a clinically suspected patient. Instead, the timing of presentation and the biological window detected by each assay should be considered.

## Conclusions

ST was frequently detected among patients presenting with acute undifferentiated fever at a tertiary-care hospital in Western Odisha. IgM ELISA and TSA-56 qPCR demonstrated different patterns of positivity, with qPCR detecting infection in a proportion of ELISA-negative patients and yielding negative results in a subset of ELISA-positive patients.

These findings highlight an important diagnostic principle: serological and molecular assays assess different biological components and stages of ST infection and should not necessarily be viewed as interchangeable tests. In clinically suspected patients, particularly those presenting early in the course of illness, combining serological and molecular approaches may provide complementary diagnostic information and improve case detection.
